# Anaerobic Digestion in the 21st Century

**DOI:** 10.3390/bioengineering7040157

**Published:** 2020-12-07

**Authors:** Marcell Nikolausz, Jörg Kretzschmar

**Affiliations:** 1 Department of Environmental Microbiology, Helmholtz Centre for Environmental Research-UFZ, Permoserstrasse 15, 04318 Leipzig, Germany; 2 Department of Biochemical Conversion, DBFZ Deutsches Biomasseforschungszentrum gemeinnützige GmbH, Torgauer Strasse 116, 04347 Leipzig, Germany; Joerg.Kretzschmar@dbfz.de

Despite being a mature biotechnological process, anaerobic digestion is still attracting considerable research attention, mainly due to its versatility both in substrate and product spectra, as well as being a perfect test system for the microbial ecology of anaerobes. This Special Issue highlights some key topics of this research field.

Anaerobic digestion (AD) originally refers to biomass degradation under anoxic conditions in both natural and engineered systems. AD is one of the oldest biotechnologies used to produce an energy carrier, i.e., biogas, from organic waste. Therefore, it can be considered as one of the earliest approaches to a circular bioeconomy. Technological development was sparse until the beginning of the 20th century; however, with increasing industrial interest, research into anaerobic microbial processes was also intensified with the aim of identifying the important process parameters and to promote methane production from all kinds of residual organic matter, especially agricultural residues such as manure and slurry. Technological progress has been made, particularly with the development of the UASB reactor at the end of 1970s [1], which facilitated AD of substrates with a very low content of total solids such as municipal or industrial wastewater (reviewed in this Special Issue by Mainardis et al. [2]). From the industrial perspective of electrical power production, with 19 GW installed capacity worldwide and 6,586 TWh electrical power production in 2018, biogas plants are major players even if not reaching the podium of top three renewables. The majority of biogas plants are located in Asia (40%), Europe (20%) and North America (19%).

Extending the definition of AD, in addition to solid and liquid substrates, it can also convert gases rich in hydrogen and carbon dioxide into methane via hydrogenotrophic methanogenesis. This pathway can be used for biological upgrading of biogas, as reviewed in this Special Issue by Adnan and co-workers [3]. The resulting methane can substitute natural gas, which opens new opportunities by a direct link to traditional petrochemistry. Due to the wide substrate spectrum, AD is an ideal end-of-pipe technology for waste treatment and energy recovery in several (bio)technological applications. Furthermore, AD can be coupled with emerging biotechnological applications, such as microbial electrochemical technologies or the production of medium chain fatty acids by anaerobic fermentation. Ultimately, because of the wide range of applications, AD is still a very vital field in science. This is impressively shown by the number of scientific publications in 2019, which has been, with more than 2800 publications, the year with the most contributions in this field since the beginning of records in 1945. In the last five years, 12,529 papers on AD were published, accounting for 49.54 % of the total publications on that topic (Web of Science, www.webofknowledge.com, accessed 05.11.2020).

However, today, the AD sector faces new challenges, such as limited feedstock availability at increased price, the reduction of subsidies as well as the low competitiveness of the current products. Therefore, the techno-economical assessment of current and future technologies is important for investors in the waste management sector, which is addressed by Bhatt and Tao in this issue [4]. To avoid competition with food and feed production, the AD feedstock spectrum is currently being extended to waste products either rich in recalcitrant lignocellulose or containing inhibitory substances such as ammonia (see the studies of Wedwitschka et al. [5] and Mahato et al. [6] in this Special Issue). The development and evaluation of various pretreatment technologies for lignocellulosic biomass is a hot topic of AD research that several articles in this Special Issue deal with (see the studies of Müller et al. [7], Schumacher et al. [8], and Monlau et al. [9]). The effect of the inoculum on the microbial community structure and performance of the AD process is still an enigma. The study of Moestedt et al. [10] in this issue sheds some light on the microbiology of process inoculation and start-up, which was handled as a black box in the past. Although academic knowledge about the microbiome, the engine driving the AD process, has been accumulating, the use of this knowledge for the innovation of AD technologies is still scarce. With the rapid development of novel sequencing technologies, we also expect changes on that and the emergence of new reactor systems or technology concepts based on ecological knowledge in the future.

The fate of veterinary antibiotics, microorganisms resistant to antibiotics, resistance genes and pathogenic microorganisms in AD is a further important topic due to the massive application of antibiotics in livestock farming (see the studies of Hosseini Taleghani et al. [11] and Russel at al. [12]). We see AD plants more as treatment options than a threat, when the process parameters are properly adjusted to maximize attenuation. Aquacultures are also hotspots of direct antibiotics usage or indirect input from untreated manure and human wastes that are still applied in many Asian shrimp and tilapia farms. In general, the sludge from aquacultures is a very specific and problematic waste but AD technology can also contribute to its treatment (an example of reactor system development is presented in this issue by Chiumenti and co-workers [13]).

Germany is one of the European leaders in biogas technology, with regards to the number of large-scale plants and their installed capacity, partially due to the generous subsidy system of the German energy transition (Renewable Energy Sources Act). However, this support has gradually decreased in recent years. This situation, in addition to comparably high feedstock prices, enhances the competition with other renewables. Otherwise, AD plants are able to provide power on demand, thus balancing the fluctuations in power generation from wind turbines and photovoltaics. Therefore, the AD plants of the 21st century should be more flexible in terms of power generation, the substrate as well as the product spectra.

All of these examples highlight that there is still an enormous potential in AD to be an important engine of new biorefinery concepts and renewable power generation and to contribute substantially to greenhouse gas reduction as well as to a circular bioeconomy.
